# Ectoine attenuates H_2_O_2_-Induced cellular senescence in human keratinocytes and endothelial cells by modulating the p53/p21 and p16 pathways

**DOI:** 10.3389/fragi.2026.1754569

**Published:** 2026-02-26

**Authors:** Meini Li, Jingyue Zhang, Wenke Yang, Zengqiang Miao, Yanran Pai, Yi Yi, Jie Shuang, Xiang Gao, Yongzhen Li

**Affiliations:** 1 Department of Basic Medical Sciences, Qinghai University Medical College, Xining, Qinghai, China; 2 Department of Clinical Laboratory, Eighth Affiliated Hospital of Guangxi Medical University, Guigang City People’s Hospital, Guigang, Guangxi, China

**Keywords:** cellular senescence, Ectoine, HaCaT, oxidative stress, p53/p21

## Abstract

**Background:**

Ectoine ((S)-2-methyl-1,4,5,6-tetrahydropyrimidine-4-carboxylic acid) is a major compatible solute found in halophilic microorganisms from salt lakes. The anti-cellular senescence effect and skin safety of Ectoine on H_2_O_2_-induced oxidative stress senescence in HaCaT cells and EA. hy926 endothelial cells were evaluated through a series of *in vitro* assays.

**Methods:**

An oxidative stress senescence model was established using H_2_O_2_ in HaCaT and EA. hy926 cells pretreated with various concentrations of Ectoine. Cell viability was assessed using the CCK-8 assay, proliferative capacity was evaluated with the EdU assay, and senescence status was determined by SA-β-gal staining. Intracellular ROS levels were measured using a DCFH probe, and cell death was analysed by flow cytometry. The expression of senescence-related markers was evaluated at the transcriptional and protein levels: The mRNA levels of *TP53*, *CDKN1A* (encoding p21), *CDKN2A* (encoding p16), *MMP2*, and *MMP9* were measured by qRT‒PCR, while their corresponding protein products (p53, p21, and p16) were analysed by Western blotting. Lamin B1 expression was examined by immunofluorescence.

**Results:**

Exposure to H_2_O_2_ successfully induced cellular senescence, as evidenced by increased SA-β-gal activity, elevated ROS levels, and upregulated expression of senescence-associated markers (*TP53, CDKN1A*, *CDKN2A*, *MMP2*, and *MMP9*), along with decreased Lamin B1 expression. Ectoine pretreatment significantly attenuated these senescence phenotypes in a concentration-dependent manner, with 0.50 μmol/L identified as the most effective concentration. At this dosage, Ectoine enhanced cell viability, reduced ROS accumulation, and suppressed cell death without causing cytotoxicity. Mechanistically, Ectoine downregulated the expression of the p53/p21 and p16 pathway components, thereby inhibiting cell cycle arrest.

**Conclusion:**

Ectoine exerts potent anti-senescence effects in H_2_O_2_-induced models of skin-related cell senescence, primarily by modulating the p53/p21 and p16 signalling pathways and reducing oxidative damage. These findings may support further exploration of its potential application in anti-cellular senescence research.

## 1 Introduction

As people age, concern regarding skin condition increases, and skin wrinkles represent a typical ageing-related phenomenon. The ageing process of skin can be divided into intrinsic ageing and extrinsic ageing (Krutmann et al., 2021). Cellular senescence, a key contributor to ageing, can be triggered by various stressors, including reactive oxygen species (ROS) and environmental factors (Tubbs and Nussenzweig, 2017; Jackson and Bartek, 2009). Studies on many senescence biomarkers and related mechanistic pathways associated with skin failure have been conducted in animal and *in vitro* models. Skin-related cells (e.g., keratinocytes) exposed to UVB light exhibit DNA damage and cell cycle arrest and express senescence biomarkers such as increased senescence-associated β-galactosidase (SA-β-Gal) activity; *TP53*, *CDKN1A*, and *CDKN2A* activation; and downregulation of Lamin B1 expression (Csekes and Račková, 2021). The expression of p16 is rapidly upregulated following ultraviolet irradiation and in response to H_2_O_2_-induced oxidative stress in a p38 stress-activated protein kinase-dependent manner (Krutmann et al., 2021). Several natural plant extracts, such as rhizocortin, gallic acid, and fexofenone, have been reported to have inhibitory effects on β-galactosidase activity, ROS levels, and p53 and p16 expression in human skin keratinocytes, thereby slowing ageing (Nisticò et al., 2015; Shin et al., 2014; Seo and Jeong, 2015).

Ectoine, a compatible solute, was first identified and structurally characterized in the saline photosynthetic violet bacterium *Salmonella* salina (*Ectothiorhodospira halochloris*) in 1985 (Galinski et al., 1985), and Rakesh et al. (Harishchandra et al., 2010) reported that the preferential exclusion model for the protection of macromolecules is based on the following mechanism: Ectoine protects affected cell membranes from desiccation-induced responses and subsequent inflammatory reactions by forming a water shell (Ectoine-water complexes) around the protein through a “preferential hydration” effect. In addition, increased hydration of cell membranes improves the fluidity and functionality of the lipid layer, and Ectoine promotes the formation of water molecule clusters; therefore, the preferential exclusion model attributes the stabilizing effect of Ectoine to changes in the surrounding water structure, which not only maintains the intra- and extracellular osmotic pressure but also increases the water content. Ectoine not only maintains intra- and extracellular osmotic pressure, increasing resistance to extreme conditions and reducing transdermal water loss, but also has a cell-stabilizing effect by protecting whole cells and biomolecules (extracellular polysaccharides, enzymes, DNA, antibodies, etc.) under harsh environmental conditions (e.g., high temperature, low temperature, or dryness) (Held and Sadowski, 2016). It also protects cells from oxidative damage (Hseu et al., 2020) and has good stress protection, functional maintenance and anti-inflammatory properties (Czech et al., 2018), and recent studies have reported that Ectoine can be used as an additive in skincare products such as skin whitening, moisturizing, antiaging, and UV protection products (Hseu et al., 2020; Bownik and Stępniewska, 2015; Bownik and Stępniewska, 2016); therefore, Ectoine has very good potential for use in biomedical development and other fields.

HaCaT cells are immortalized cell lines derived from the spontaneous transformation of human skin keratinocytes, and their physiological properties are very similar to those of skin keratinocytes, which have long been regarded as reliable and stable cell lines in the field of skin research (Zhang et al., 2023). EA. hy926 endothelial cells, as a fusion line of human umbilical vein endothelial cells (HUVECs), are more likely to be HUVECs and are easier to culture *in vitro*; therefore, these cells have been widely adopted by researchers in recent years (Chavez et al., 2024).

In this study, we investigated the potential anti-senescence efficacy of Ectoine on skin-related cells using human immortalized keratinocytes (HaCaT cells) and human umbilical vein endothelial cells (EA.hy926) to establish an oxidative stress-induced cellular senescence model. We explored the effects of Ectoine on oxidative stress-induced senescent skin-related cells related to the Lamin B1, ROS, p53/p21 and p16 pathways.

## 2 Materials and methods

### 2.1 Materials and cell lines

Ectoine was obtained from Damas-beta (Cat No. 011009959 91759B). HaCaT cells were purchased from Shanghai Fuheng Biotechnology Co., Ltd. (Cat No. FH0186). EA. hy926 cells were kindly provided by the Antitumour Group of Chinese and Tibetan Medicines (CTmat, Qinghai University, China).

### 2.2 Cell culture and general experimental procedures

#### 2.2.1 Cell culture and treatment protocol

Cells were seeded at a density of 2 × 10^5^ cells/mL in appropriate culture plates. In 96-well plates, 100 μL of cell suspension was added to each well, with peripheral wells filled with 100 μL of PBS to minimize edge effects. To 6-well plates, 1 mL of cell suspension was added per well. After approximately 60% confluence was reached, the cells were grouped according to the experimental design and treated as specified in the subsequent sections. All the cells were maintained at 37 °C in a 5% CO_2_ incubator.

#### 2.2.2 Cytotoxicity assessment

Cell viability was determined using the CCK-8 assay. Following treatment with Ectoine (0.25–2 μmol/L) or H_2_O_2_ (100–400 μmol/L) as described in the respective experimental sections, 10 μL of CCK-8 solution was added to each well and incubated for 0.5 h. Absorbance was measured at 450 nm using a microplate reader.

### 2.3 EdU proliferation assay

Cell proliferation was assessed using an EdU cell proliferation kit according to the manufacturer’s instructions. Briefly, cells were treated as specified, incubated with EdU solution for 2 h, fixed, and stained with Apollo^®^ dye. The nuclei were counterstained with Hoechst 33342. Images were acquired using a fluorescence microscope, and proliferation rates were calculated as the ratio of EdU-positive cells to total cells using ImageJ software.

### 2.4 Senescence-associated β-galactosidase staining

Cellular senescence was detected using an SA-β-gal staining kit according to the manufacturer’s protocol. After treatment, the cells were fixed with fixative solution for 15 min at room temperature and incubated with staining solution overnight at 37 °C without CO_2_. Senescent cells were identified by blue staining under a light microscope.

### 2.5 Intracellular ROS measurement

Intracellular ROS levels were measured using a DCFH-DA fluorescent probe. Following treatment, the cells were incubated with 10 μmol/L DCFH-DA at 37 °C for 20 min and then were washed with PBS, after which the fluorescence intensity was immediately measured using a fluorescence microscope. Quantitative analysis was performed using ImageJ software.

### 2.6 Quantitative real-time PCR

Total RNA was extracted using TRIzol reagent, and cDNA was synthesized using the PrimeScript RT reagent kit. Quantitative PCR was performed using SYBR Green Premix on a real-time PCR system. Gene expression levels were normalized to those of β-actin and calculated using the 2^(-ΔΔCt)^ method. The primer sequences used in this study are listed in Supplementary Table S1.

### 2.7 Western blot analysis

Total protein was extracted using RIPA lysis buffer, and the protein concentration was determined using a BCA assay. The proteins were separated by SDS‒PAGE and transferred to PVDF membranes. After blocking with 5% nonfat milk, the membranes were incubated with primary antibodies against p53, p21, p16 and β-actin overnight at 4 °C, followed by incubation with HRP-conjugated secondary antibodies. The protein bands were visualized using the ECL substrate and quantified using ImageJ software.

### 2.8 Immunofluorescence staining

Cells grown on coverslips were fixed with 4% paraformaldehyde, permeabilized with 0.1% Triton X-100, and blocked with 5% BSA. The samples were incubated with primary antibody against Lamin B1 overnight at 4 °C, followed by incubation with a fluorescent secondary antibody. The nuclei were counterstained with DAPI, and images were captured using a fluorescence microscope.

### 2.9 Apoptosis analysis

Apoptosis was detected using an Annexin V-FITC/PI apoptosis detection kit according to the manufacturer’s instructions. After treatment, the cells were collected, resuspended in binding buffer, and stained with Annexin V-FITC and PI for 15 min at room temperature in the dark. Apoptotic rates were analysed by flow cytometry within 1 h.

### 2.10 Statistical analysis

The data are presented as the mean ± standard deviation (SD) of three independent biological replicates (n = 3). The normality of the data distribution was assessed using the Shapiro–Wilk test. Differences between groups were analysed by one-way analysis of variance (ANOVA), followed by Tukey’s *post hoc* test for multiple comparisons. A value of *P* < 0.05 was considered to indicate statistical significance. All the statistical analyses were performed using SPSS 22.0 (IBM Corp., USA). The graphs were generated using GraphPad Prism 9.

## 3 Results

### 3.1 Cytotoxicity and morphology assessment

**FIGURE 1:**
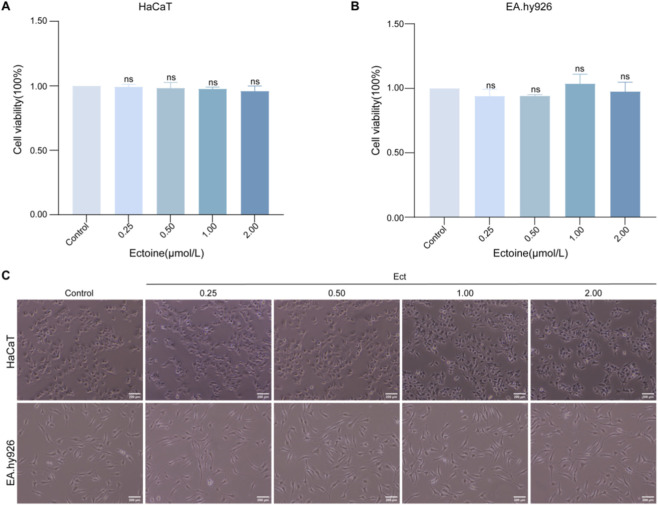
Evaluation of Ectoine cytotoxicity and its effects on cell morphology. **(A,B)** Viability of HaCaT and EA. hy926 cells treated with Ectoine (0.25–2 μmol/L) for 24 h, as determined by the CCK-8 assay (n = 3 independent biological replicates). **(C)** Morphology of HaCaT and EA. hy926 cells following 24 h of Ectoine treatment.

To evaluate the biosafety profile of Ectoine, its potential cytotoxicity to HaCaT keratinocytes and EA. hy926 endothelial cells was assessed via a CCK-8 assay after 24 h of treatment. As demonstrated in Figures 1A,B, compared with control treatment, treatment with Ectoine at concentrations ranging from 0.25 to 2 μmol/L did not significantly decrease cell viability in either cell type (*P* > 0.05; n = 3), indicating that Ectoine is not cytotoxic within this dose range. Morphological observations further corroborated these findings, revealing that compared with control cells, Ectoine-treated cells retained normal cellular architecture without observable anomalies (Figure 1C). Notably, the Ectoine-treated groups exhibited increased cell density under microscopic evaluation, suggesting a potential pro-proliferative effect that warranted further investigation via an EdU assay.

### 3.2 Establishment of an H_2_O_2_-induced senescence model

**FIGURE 2:**
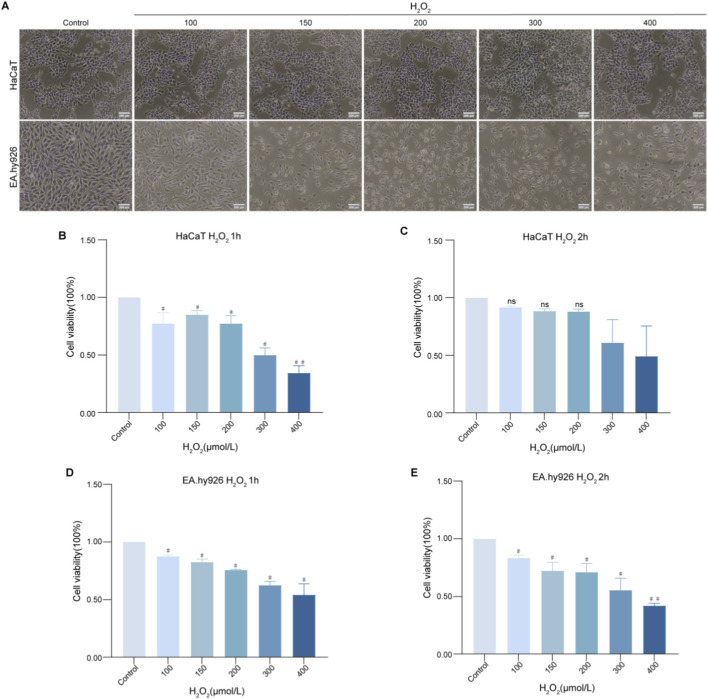
Effect of H_2_O_2_ on the viability of HaCaT and EA. hy926 cells. **(A)** Morphology of HaCaT cells treated with 100–400 μmol/L H_2_O_2_ for 1 h and EA. hy926 cells treated with the same concentration range for 2 h (×20 magnification). **(B–E)** Viability of **(B,C)** HaCaT and **(D,E)** EA. hy926 cells after H_2_O_2_ exposure for 1 h or 2 h. The data are presented as the mean ± SD (n = 3 independent biological replicates). #*P* < 0.05, ##*P* < 0.01 vs. the control group.

To establish a model of oxidative stress-induced senescence, HaCaT and EA. hy926 cells were exposed to H_2_O_2_ across a concentration gradient of 100–400 μmol/L for one or 2 h. This treatment induced discernible morphological alterations in both cell lines, such as a reduction in the cell number (Figure 2A). As illustrated in Figures 2B–E, H_2_O_2_ treatment induced a concentration-dependent suppression of viability in both cell lines. On the basis of the conventional senescence criterion corresponding to approximately 70%–80% cell viability, the conditions of 200 μmol/L H_2_O_2_ for 1 h (resulting in 87.85% viability) and 100 μmol/L H_2_O_2_ for 2 h (86.10% viability) were selected to induce senescence in HaCaT and EA. hy926 cells, respectively, and these conditions were used in all subsequent experiments.

### 3.3 Ectoine promotes proliferation and reduces senescence-associated β-galactosidase activity

**FIGURE 3:**
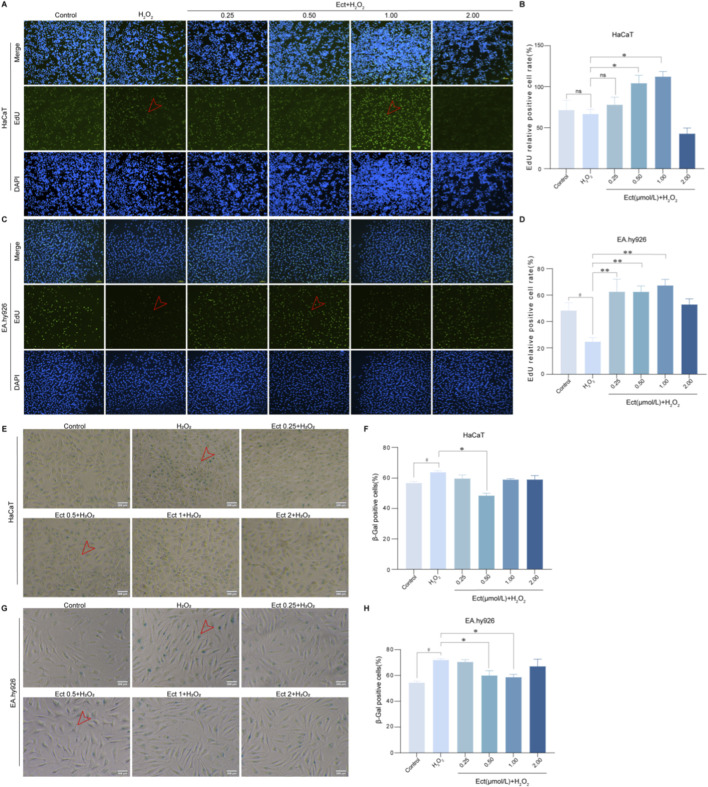
Ectoine promotes proliferation and reduces senescence in HaCaT and EA. hy926 cells. **(A–D)** An EdU assay was used to detect the proliferation of **(A,B)** HaCaT and **(C,D)** EA. hy926 cells after Ectoine treatment. **(E–H)** SA-β-gal staining (100×) of **(E,F)** HaCaT and **(G,H)** EA. hy926 cells under different treatments. Data are presented as the mean ± SD (n = 3 independent biological replicates). #*P* < 0.05 vs. the control group; *P < 0.05, **P < 0.01 vs. the H_2_O_2_ group.

To determine whether Ectoine mitigates H_2_O_2_-induced proliferative arrest, we performed EdU incorporation assays. Cells were pretreated with Ectoine for 24 h prior to H_2_O_2_ exposure under predetermined optimal senescence-inducing conditions. Quantitative analysis demonstrated that Ectoine pretreatment significantly counteracted the antiproliferative effect of oxidative stress. As shown in Figures 3A–D, compared with H_2_O_2_, 0.5 and 1 μmol/L Ectoine markedly increased the percentage of EdU-positive HaCaT and EA. hy926 cells (*P* < 0.05; n = 3), indicating effective restoration of proliferation capacity. This dose-dependent increase in DNA synthesis highlights the role of Ectoine in facilitating cellular replication under oxidative stress.

Consistent with the senescent phenotype, compared with the control treatment, H_2_O_2_ challenge significantly increased the proportion of SA-β-gal-positive HaCaT (63.89%, *P* < 0.05; n = 3) and EA. hy926 (72.00%, *P* < 0.05; n = 3) cells (Figures 3E–H). Notably, pretreatment with 0.5 and 1 μmol/L Ectoine substantially attenuated this H_2_O_2_-induced increase in SA-β-gal activity (*P* < 0.05), demonstrating its strong ability to ameliorate stress-induced premature senescence.

### 3.4 Ectoine reduces intracellular ROS levels and preserves Lamin B1 expression

**FIGURE 4:**
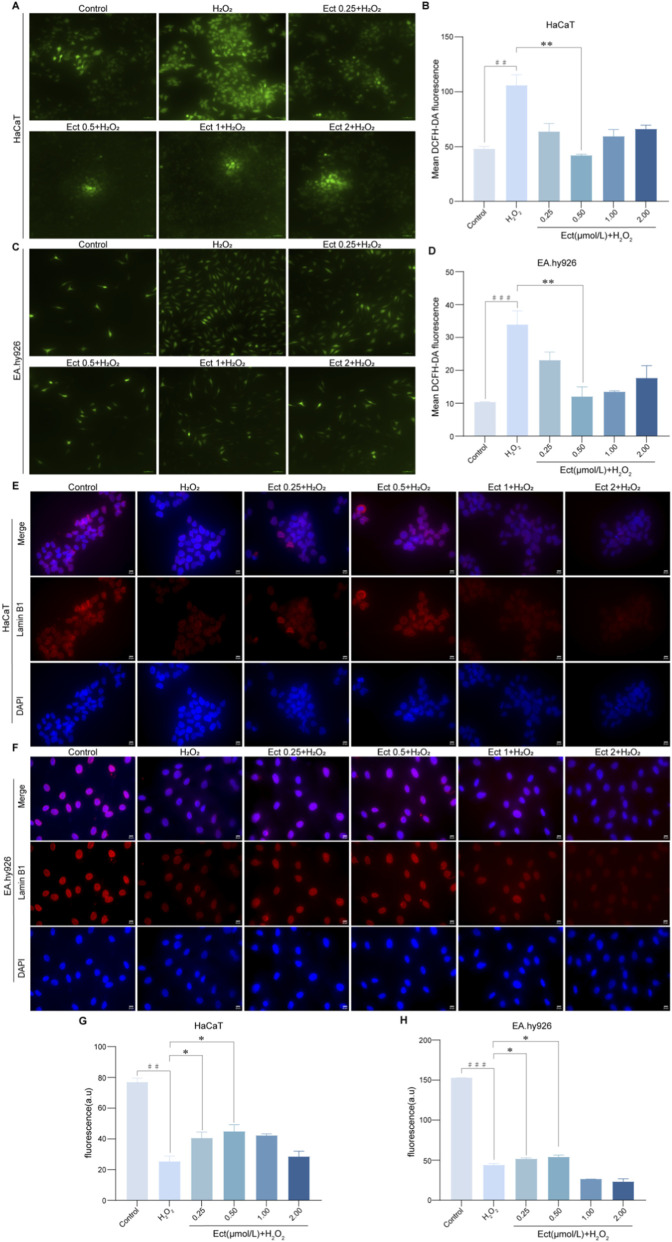
Ectoine attenuates intracellular ROS levels and maintains Lamin B1 expression in HaCaT and EA. hy926 cells. **(A–D)** Intracellular ROS levels detected by a DCFH-DA fluorescent probe in **(A,B)** HaCaT and **(C ,D)** EA. hy926 cells. **(E–H)** Lamin B1 expression detected by immunofluorescence in **(E,F)** HaCaT and **(G,H)** EA. hy926 cells. The data are presented as the mean ± SD (n = 3 independent biological replicates). ##*P* < 0.01, ###*P* < 0.001 vs. the control group; *P < 0.05, **P < 0.01 vs. the H_2_O_2_ group.

We next investigated the antioxidant potential of Ectoine by measuring intracellular ROS levels following H_2_O_2_-induced oxidative insult. As depicted in Figures 4A–D, H_2_O_2_ exposure induced substantial ROS accumulation in both cell types. However, pretreatment with 0.5 μmol/L Ectoine significantly attenuated this oxidative burst, reducing ROS levels to levels comparable to those in the control groups (*P* < 0.05; n = 3). These results indicate that Ectoine effectively quenches oxidative stress in both keratinocytes and endothelial cells.

Given that loss of nuclear membrane integrity is a hallmark of senescence, we assessed Lamin B1 expression via immunofluorescence. Compared with control treatment, H_2_O_2_ treatment significantly reduced the Lamin B1-to-DAPI fluorescence ratio (*P* < 0.01 for HaCaT; *P* < 0.001 for EA. hy926; n = 3), indicating disruption of nuclear envelope integrity. Ectoine pretreatment (0.25 and 0.5 μmol/L) significantly restored Lamin B1 expression (*P* < 0.05 vs. the H_2_O_2_ group), suggesting that it plays a role in preserving nuclear architecture and mitigating DNA damage response activation (Figures 4E–H).

### 3.5 qRT‒PCR analysis of senescence-related gene expression

**FIGURE 5:**
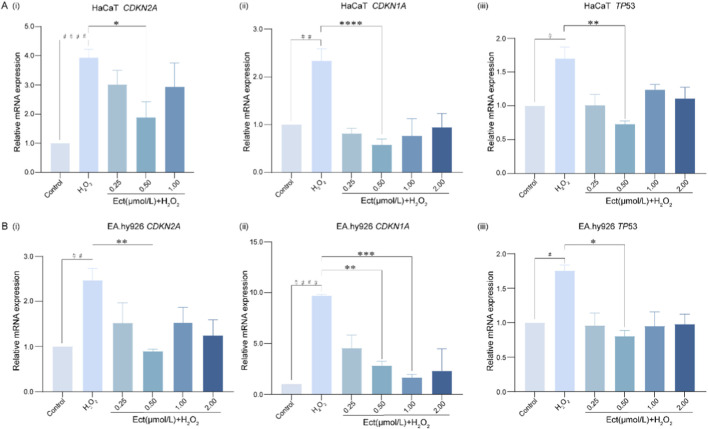
Ectoine downregulates senescence-related gene expression in H2O2-induced HaCaT and EA. hy926 cells. The data are presented as the mean ± SD (n = 3 independent biological replicates). (A) Relative mRNA expression levels of CDKN2A, CDKN1A, and TP53 in HaCaT cells. (i) CDKN2A, (ii) CDKN1A, (iii) TP53. (B) Relative mRNA expression levels of the same genes in EA.hy926 cells. (i) CDKN2A, (ii) CDKN1A, (iii) TP53. ^#^ *P* < 0.05, ^##^ *P* < 0.01, ^####^ *P* < 0.0001 vs. the control group; *P < 0.05, **P < 0.01, ***P < 0.001, ****P < 0.0001 vs. the H_2_O_2_ group.

qRT‒PCR analysis revealed that H_2_O_2_-induced oxidative stress significantly upregulated the mRNA expression of pivotal senescence-related genes (*TP53, CDKN1A* and *CDKN2A*) in both cell lines. As illustrated in Figure 5, Ectoine pretreatment effectively suppressed this H_2_O_2_-triggered upregulation. Specifically, 0.5 μmol/L Ectoine most potently downregulated mRNA expression in HaCaT cells, while in EA. hy926 cells, 0.5–1 μmol/L Ectoine most effectively suppressed mRNA expression (all *P* < 0.05, n = 3), indicating effective inhibition of core senescence pathways under oxidative stress.

Given that ROS-driven extracellular matrix (ECM) degradation is a key feature of cellular senescence, we examined the effects of Ectoine on the matrix metalloproteinases MMP2 and MMP9. H_2_O_2_ exposure significantly upregulated MMP9 mRNA expression in both cell types and MMP2 expression in EA. hy926 cells (*P* < 0.05; n = 3). Conversely, in EA. hy926 cells, 0.5 μmol/L Ectoine significantly suppressed both MMP2 and MMP9 expression (*P* < 0.05; n = 3), suggesting that cell type-specific regulation of ECM degradation occurred (Supplementary Figure S1).

### 3.6 Ectoine downregulates senescence-related protein expression and inhibits apoptosis

**FIGURE 6:**
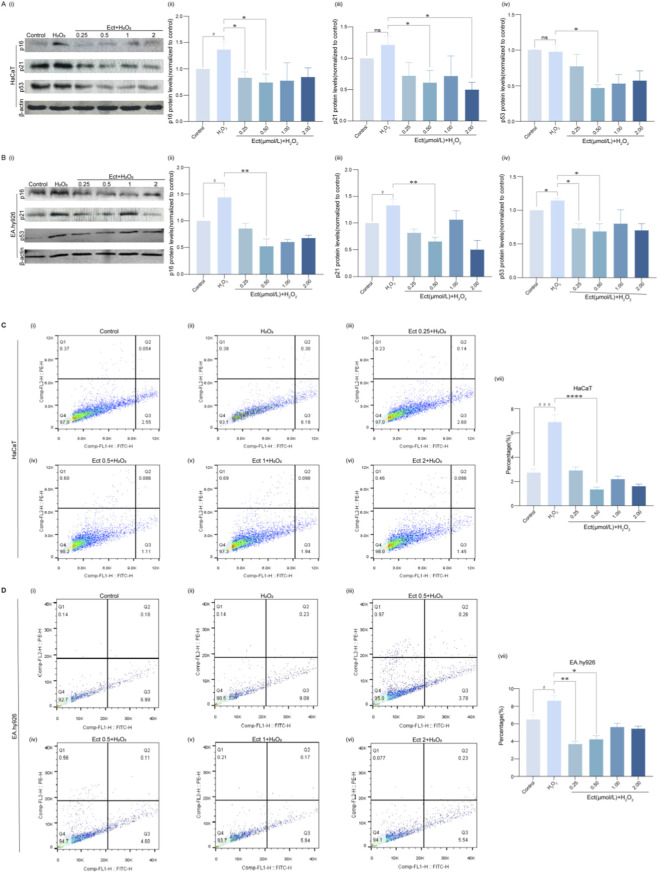
Ectoine downregulates senescence-related protein expression and reduces apoptosis in HaCaT and EA. hy926 cells. **(A,B)** Western blot analysis of p16, p21, and p53 protein expression in **(A)** HaCaT and **(B)** EA. hy926 cells. **(C,D)** Apoptosis rate detected by flow cytometry in **(C)** HaCaT and **(D)** EA. hy926 cells. The apoptosis rate was calculated as the sum of early (lower right quadrant) and late (upper right quadrant) apoptotic cells. The data are presented as the mean ± SD (n = 3 independent biological replicates). ^#^ *P* < 0.05, ^###^ *P* < 0.001 vs. the control group; *P < 0.05, **P < 0.01, ****P < 0.0001 vs. the H_2_O_2_ group.

Western blot analysis confirmed the regulatory effects of Ectoine at the protein level. While H_2_O_2_ treatment tended to increase p16, p21, and p53 protein expression, Ectoine pretreatment significantly reversed this increase. In both cell lines, 0.5 μmol/L Ectoine most effectively suppressed protein expression (Figures 6A,B; *P* < 0.05; n = 3), demonstrating that Ectoine effectively suppressed senescence-related protein expression under oxidative stress conditions.

Flow cytometric analysis using Annexin V-FITC/PI staining indicated that H_2_O_2_ significantly increased apoptosis in HaCaT (6.48% vs. 2.60% in control, *P* < 0.001; n = 3) and EA. hy926 (8.69% vs. 6.42%; *P* < 0.05; n = 3) cells. Ectoine pretreatment (0.5 μmol/L) significantly attenuated H_2_O_2_-induced apoptosis in a concentration-dependent manner (*P* < 0.0001 for HaCaT cells; *P* < 0.05 for EA. hy926 cells; n = 3), confirming its protective effect against oxidative stress-induced cell death (Figures 6C,D).

## 4 Discussion

In this study, we demonstrated that H_2_O_2_ treatment successfully induced cellular senescence in both HaCaT keratinocytes and EA. hy926 endothelial cells, as indicated by increased SA-β-gal activity, elevated intracellular ROS levels, upregulated expression of senescence-related markers (*TP53*, *CDKN1A*, *CDKN2A*, *MMP2*, and *MMP9*) and reduced Lamin B1 expression. Pretreatment with Ectoine, particularly at 0.50 μmol/L, significantly attenuated these senescence-associated changes in a concentration-dependent manner.

Cellular senescence can be triggered by oxidative stress, such as exposure to H_2_O_2_, leading to irreversible cell cycle arrest and characteristic phenotypic changes (Pezone et al., 2023). In the present oxidative stress-induced cellular senescence model, the increase in SA-β-gal activity and the upregulation of senescence markers confirmed the successful establishment of cellular senescence. Ectoine pretreatment markedly reduced SA-β-gal positivity and suppressed the expression of p53, p21, p16 and MMPs while preserving Lamin B1 levels. These results indicate that Ectoine effectively counteracts H_2_O_2_-induced premature senescence in skin-related cells.

Excessive ROS are key drivers of senescence. Studies have shown that natural compounds such as mangiferin can delay H_2_O_2_-induced senescence in skin-related cells, including HaCaT and HSF cells, by modulating ROS and MMP levels (Seo and Jeong, 2015; Kanoi et al., 2021). In our model, Ectoine pretreatment significantly decreased intracellular ROS levels and reduced apoptosis in both cell types. Notably, while apoptosis and senescence represent distinct cellular outcomes, the suppression of H_2_O_2_-induced apoptosis by Ectoine may contribute to overall cell survival under oxidative stress conditions. The more pronounced antiapoptotic effect observed in HaCaT cells suggests cell type-specific responses to Ectoine treatment.

The p53/p21 and p16 pathways are central regulators of cellular senescence. *TP53*, activated by DNA damage, induces cell cycle arrest primarily through its downstream target *CDKN1A*, while *CDKN2A* contributes to sustained proliferative arrest (Pezone et al., 2023). Our data revealed that Ectoine downregulated the mRNA and protein expression of p53, p21, and p16 in H_2_O_2_-treated cells. On the basis of the current evidence, we propose two nonexclusive mechanistic possibilities: Ectoine may act upstream by reducing initial oxidative DNA damage through its antioxidant properties or downstream by suppressing senescence signalling pathways after damage has occurred. The observed reduction in ROS levels supports potential upstream effects, whereas the modulation of p53/p21 and p16 expression is consistent with downstream effects. We propose that our findings represent a “mechanism association” rather than direct mechanistic proof. Future work must discern whether the protective effect of Ectoine occurs upstream via prevention of DNA damage mediated by ROS or downstream via modulation of the p53/p16 pathway by combining siRNA and γH2AX/53BP1 foci analysis.

Notably, Ectoine has demonstrated significant barrier-protective and anti-inflammatory effects in various physiological models, including intestinal inflammation.​ Its underlying mechanisms—such as inhibiting the activation of classical proinflammatory signalling pathways (e.g., NF-κB and MAPK) and reducing the levels of key proinflammatory mediators (Abdel-Aziz et al., 2013; Castro-Ochoa et al., 2019)—closely overlap with​ intracellular signalling cascades triggered by skin photoaging and oxidative stress. The results of the present study confirm that Ectoine effectively mitigates H_2_O_2_-induced senescence in skin-related cells, a benefit likely rooted in its broad regulatory capacity over core inflammatory and stress pathways. Therefore, the barrier-repairing and anti-inflammatory properties observed in intestinal models provide strong theoretical support and novel research insights for the application of Ectoine in skin-related cell antiaging strategies, particularly those targeting inflammatory ageing and compromised skin barrier function. While our study provides evidence for the effects of Ectoine on key senescence markers through MMP2/9 analysis, future investigations should include protein-level validation of MMP activity and broader SASP profiling (e.g., of IL-6, IL-8, and CXCLs) to fully characterize the senescence-associated secretory phenotype. The cell-type-specific variation in Ectoine efficacy between HaCaT and EA. hy926 cells may be attributed to differences in stress response mechanisms or pathway activation, warranting further investigation. Additionally, the relationship between oxidative stress and other cell death mechanisms, such as ferroptosis, merits attention. Recent studies using multilevel experimental validation approaches, including cell models and *in vivo* systems, have demonstrated the role of ferroptosis in ageing and damage models (Yuan et al., 2025), providing a broader context for understanding the potential protective mechanisms of Ectoine.

Ectoine is known for its ability to stabilize biomacromolecules and protect cells under extreme conditions (Hseu et al., 2020; Chen et al., 2020). Although our study focused on senescence-related gene expression, the underlying mechanisms may involve “preferential hydration”, and interactions with signalling pathways such as ERK1/2, JNK, and P38 MAPK pathways (Cai et al., 2021) warrant further investigation. Given its anti-inflammatory and membrane-stabilizing properties, the utility of Ectoine may extend to neuroprotection, particularly in Alzheimer’s disease, where oxidative stress, protein misfolding, and inflammation are involved. Future work should employ advanced models, such as Aβ-treated neuronal cultures or transgenic Alzheimer’s models, to validate its efficacy in neural systems. In conclusion, Ectoine effectively counteracts H_2_O_2_-induced senescence in skin-related cells, supporting its potential in antiaging skincare. Moreover, its pleiotropic mechanisms, including biomacromolecule stabilization, antiaggregation, and pathway modulation, suggest promising translational value for addressing neurological ageing and related disorders, meriting expanded interdisciplinary research.

## Data Availability

The datasets presented in this study can be found in online repositories. The names of the repository/repositories and accession number(s) can be found in the article/Supplementary Material.
