# Supplementary material for: Ectoine attenuates H2O2-Induced cellular senescence in human keratinocytes and endothelial cells by modulating the p53/p21 and p16 pathways
Source: Front Aging. 2026 Feb 26;7:1754569. doi: 10.3389/fragi.2026.1754569 (PMC12979466; doi:10.3389/fragi.2026.1754569)
Supplement: Supplementary file 1 [file Supplementaryfile1.docx]

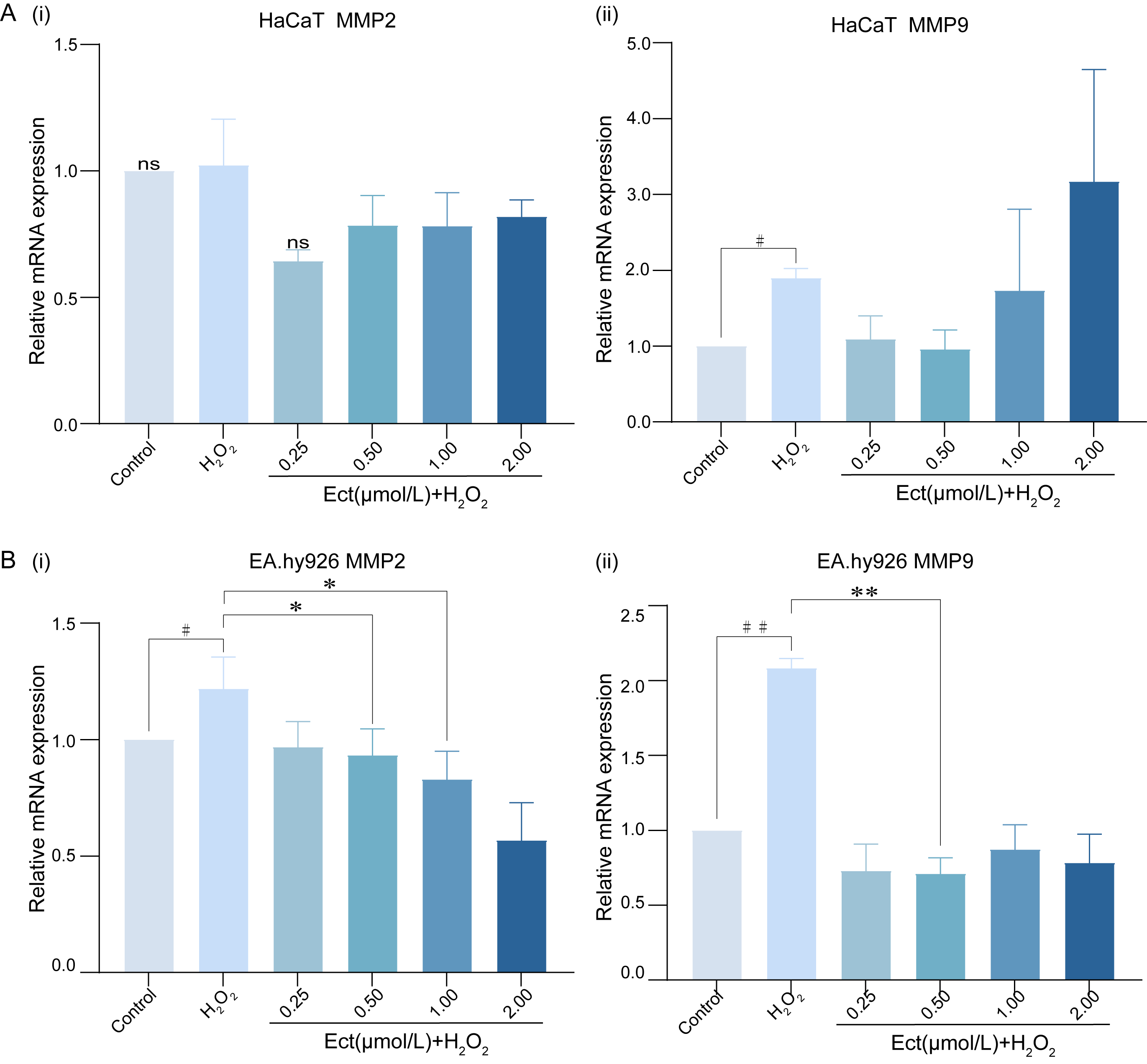


Fig. S1 Ectoine downregulates MMPs gene expression in H₂O₂-induced HaCaT and EA.hy926 cells. Data are presented as mean ± SD (n=3 independent biological replicates). ^#^*P* < 0.05,^##^*P* < 0.01 vs. Control group; **P* < 0.05, ***P* < 0.01 vs. H₂O₂ group.

**Table S1** Details of qPCR primer sequences

| Gene Name | Forward Sequence | Reverse Sequence |
| --- | --- | --- |
| GAPDH | GAAGGTGAAGGTCGGAGT | CATGGGTGGAATCATATTGGAA |
| *p*16 | CTTCCTGGACACGCTGGTG | AATCGGGGATGTCTGAGGGA |
| *p*21 | CACCACTGGAGGGTGACTTC | ATCTGTCATGCTGGTCTGCC |
| *p*53 | AGGTTGGCTCTGACTGTACC | GATTCTCTTCCTCTGTGCGC |

**Table S2** Immunohistochemistry (IHC) and Western blot (WB) antibodies

| Antibody | Catalog number and company | Dilution |
| --- | --- | --- |
| p16 | #10883-1-AP (Proteintech) | WB 1:4000 |
| P21 | #10355-1-AP (Proteintech) | WB 1:2000 |
| p53 | #60283-2-Ig (Proteintech) | WB 1:5000 |
| β-actin | #20536-1-AP (Proteintech) | WB 1:5000 |
| Lamin B1 | #66095-1-Ig (Proteintech) | IHC 1:500 |
